# Intramolecular carbolithiation cascades as a route to a highly strained carbocyclic framework: competition between 5-*exo*-*trig* ring closure and proton transfer

**DOI:** 10.3762/bjoc.9.59

**Published:** 2013-03-14

**Authors:** William F Bailey, Justin D Fair

**Affiliations:** 1 Department of Chemistry, University of Connecticut, Storrs, Connecticut 06269-3060, USA; 2 Department of Chemistry, Indiana University of Pennsylvania, Indiana, PA 15705, USA

**Keywords:** carbolithiation cascade, carbometallation, intramolecular carbolithiation, intermolecular proton transfer, lithium–halogen exchange, strained hydrocarbons

## Abstract

The preparation of fairly strained carbocyclic ring systems by intramolecular 5-*exo*-*trig* ring closure has been well documented, and the absence of proton transfers that would compromise such cyclizations is a hallmark of this chemistry. In an effort to explore the limitations of this approach to more highly strained systems, the preparation of a stellane (tricyclo[3.3.0.0^3,7^]octane) framework by an intramolecular carbolithiation cascade involving three coupled 5-*exo-trig* cyclizations of the vinyllithium derived from 2-bromo-4-vinyl-1,6-heptadiene by lithium–bromine exchange was investigated. The cascade does not afford the stellane; rather, the cascade is terminated after two cyclizations by a proton transfer that occurs by an intermolecular process catalyzed by trace amounts of *endo*-5-methyl-2-methylenebicyclo[2.2.1]heptane present in reaction mixtures as a consequence of inadvertent quenching of an intermediate alkyllithium during prolonged reaction times at room temperature.

## Introduction

The first publication describing an intramolecular carbolithiation appeared in 1968: Drozd and co-workers reported that 5-hexenyllithium, prepared in Et_2_O by treatment of 6-bromo-1-hexene with lithium metal, cyclized at 0 °C to give (cyclopentylmethyl)lithium [1–2]. This observation was confirmed and extended in a seminal 1972 communication by John Oliver’s group [3] in which it was presciently noted that, “this reaction appears to provide an interesting … procedure for formation of five-membered ring systems which is potentially significant for synthetic purposes” [3]. Indeed, the facile cyclization of olefinic and acetylenic organolithiums has proven to be a regiospecific and highly stereoselective route [4] to a variety of functionalized carbocyclic [5–7] and heterocyclic systems [8–9].

The bonding changes that accompany cyclization of an unsaturated organolithium indicate that the process should be energetically favorable since a σ-bond (bond energy ca. 88 kcal/mol) is generated at the expense of a π-bond (bond energy ca. 60 kcal/mol). As a consequence, strained carbocyclic systems may be constructed by operationally irreversible [10] intramolecular carbolithiations [11–15]. At the outset of our foray into this area several decades ago [16], we were initially surprised to find that the cyclization of olefinic alkyllithiums was not compromised by proton transfers that would afford the more stable allyllithium isomers. A subsequent study of the behavior of 5-hexenylalkalis demonstrated that cyclization is unique to the lithium species: the Na, K, Rb, and Cs analogues of 5-hexenyllithium rearrange rapidly by [1,4]-proton transfer to afford the allylic species [17]. In fact, the absence of proton transfers that would compromise 5-*exo* cyclization of 5-hexenyllithiums is a hallmark of this chemistry. Intrigued by these observations, we were prompted to investigate the possibility of constructing a highly strained system by an intramolecular carbolithiation cascade involving three coupled 5-*exo-trig* cyclizations.

**Scheme 1:**
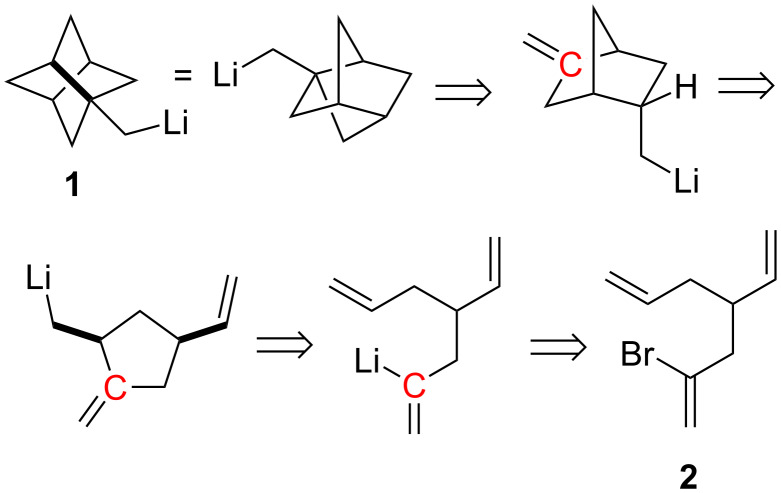
Retrosynthetic plan.

Although many strained molecules could have been selected for this exploration, the stellane framework (tricyclo[3.3.0.0^3,7^]octane [18–19]), **1**, with its mesmerizing symmetry, was chosen as the synthetic target. The retrosynthesis is depicted in Scheme 1; the stereochemical outcome anticipated for each of the ring closures finds ample literature precedent [4]. It may be noted that the nucleophilic carbon of the vinyllithium that initiates the first cyclization becomes the electrophilic carbon that terminates the cascade to give **1**.

As demonstrated by the results presented below, this cascade does not afford **1**. Rather, the cascade is terminated after two cyclizations by a proton transfer that occurs through an intermolecular process.

## Results and Discussion

**Scheme 2:**
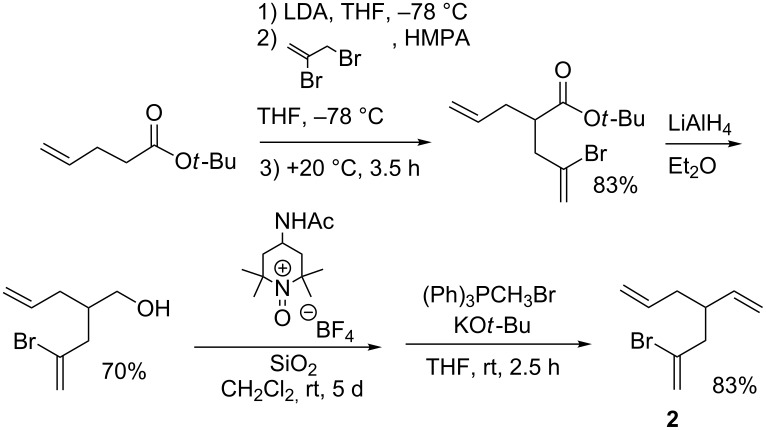
Preparation of **2**.

**Scheme 3:**
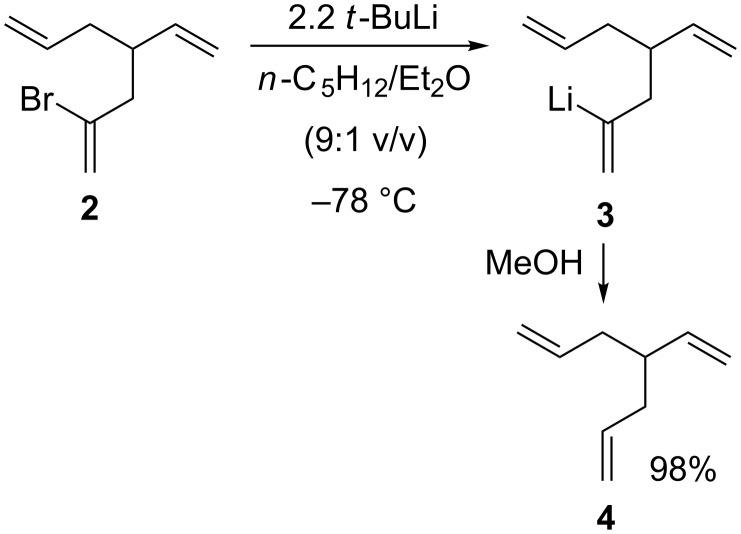
Generation of **3** by lithium–bromine exchange.

The 2-bromo-4-vinyl-1,6-heptadiene (**2**), required for the lithium–halogen exchange step that initiates the cascade, was prepared as illustrated in Scheme 2 (for details, see Supporting Information File 1). Vinyllithium **3** was cleanly generated in virtually quantitative yield at –78 °C by addition of 2.2 molar equiv of *tert*-butyllithium (*t*-BuLi) in pentane to a 0.1 M solution of **2** in *n*-pentane/diethyl ether (9:1 v/v). As would be expected, vinyllithium **3** is stable at low temperature and, as depicted in Scheme 3, quenching of a typical reaction mixture at –78 °C with oxygen-free MeOH affords 4-vinyl-1,6-heptadiene (**4**) in 98% isolated yield. Quenching with MeOD afforded an authentic sample of **4** deuterated at the C(2) position (^2^H NMR: δ 5.84–5.75 (m, 1D)); the lack of a molecular ion in the GC–MS of triene **4** precluded accurate determination of the deuterium content.

**Scheme 4:**
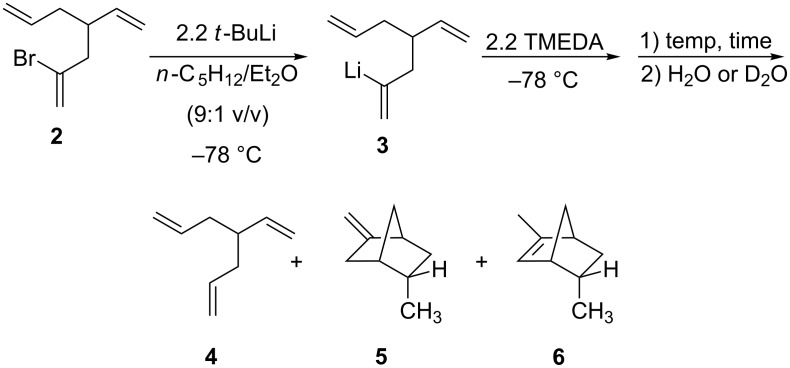
Cascade products.

Addition at –78 °C of dry, oxygen-free *N*,*N*,*N*’,*N*’-tetramethylethylenediamine (TMEDA) to solutions of **3** in *n*-C_5_H_12_/Et_2_O (9:1 v/v) and subsequent warming of the reaction mixtures for various times at several different temperatures initiated the cascades. The results of these experiments are summarized in Scheme 4 and Table 1. Crude product mixtures were analyzed by capillary GC and by GC–MS affording baseline separation of the three products (**4**–**6**), illustrated in Scheme 4, which accounted for essentially the total material balance. The structures of the bicyclic products, **5** and **6**, were established as detailed in the Experimental Section (see Supporting Information File 1 for details) by NMR and GC–MS: an authentic sample of **5** was prepared as illustrated below and **6** is a known compound [20]. It is noteworthy that no 1-methylstellane was detected as a product from any of the reactions.

**Table 1:** Intramolecular cascade results (Scheme 4).

				products, % yield^a^ (% *d*_1_)^b^		
entry	time, h	temp, °C	quench	**4**	**5**	**6**

1	1	−78 to +24	D_2_O	22.8	65.2 (62)	12.0 (75)
2	3	−78 to +24	H_2_O	4.8	63.4	31.8
3	3	−78 to +24	D_2_O	5.8	64.0 (90)	30.2 (99)
4	20.5	−78 to −40	MeOD	7.6	79.2 (94)	13.2 (84)
5	20.5	−78 to +24	D_2_O	3.2	55.0 (94)	41.8 (92)
6	8; 3	−78 to −40, +40	D_2_O	1.5	60.0 (98)	38.5 (94)

^a^Yields were determined by capillary GC. ^b^Percent (*d*_1_) deuterium incorporation determined by GC–MS.

Stirring a reaction mixture for 1 h at room temperature demonstrated that the first cyclization was not complete, as 23% of the quenched vinyllithium (**4**) remained (Table 1, entry 1). Warming reaction mixtures at room temperature for 3 h decreased the proportion of **4** (~6%); however, the yield of the norbornene product, **6**, increased from 12% after 1 h to ~30% (Table 1, entries 2 and 3). Longer reaction times at both −40 °C and +24 °C were probed to access the effect of temperature on the product distribution. Holding a reaction mixture at room temperature for 20.5 h did not favorably change the product distribution (Table 1, entry 5), while keeping a reaction at −40 °C for 20.5 h limited the amount of **6** while increasing the proportion of **5** (Table 1, entry 4). In an attempt to drive a final cyclization to give 1-methylstellane, a sample was kept at −40 °C for 8 h before being warmed to +40 °C for 3 h; the product distribution from this experiment (Table 1, entry 6) was similar to that obtained when the reaction mixture was stirred for 3 h at room temperature.

**Figure 1:**
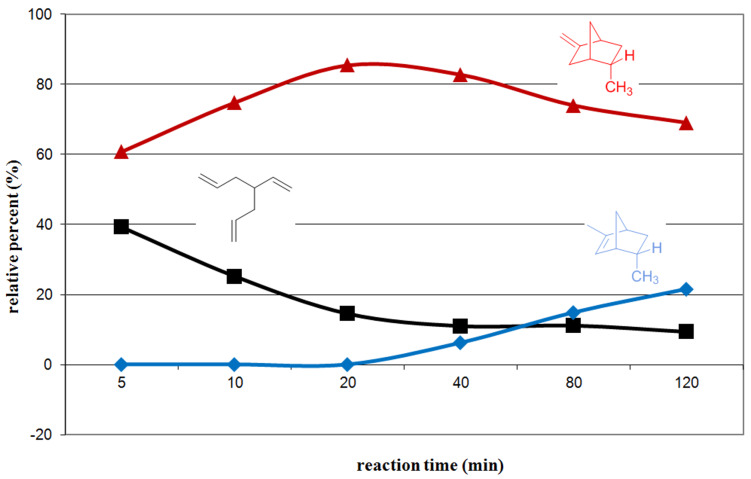
Reaction progress of the attempted triple-cyclization cascade.

In an effort to follow the progress of the reaction, product formation was monitored by removing aliquots from a reaction mixture held at room temperature and, following quenching with a mixture of diethyl ether and water, analysis of the product mixtures by capillary GC. The graph depicted in Figure 1 illustrates that the first cyclization is essentially completed after about 30 min at room temperature. Longer reaction time results in the formation of **5** and **6**; there was no evidence for the presence of 1-methylstellane. Indeed, the product distribution observed after 2 h at room temperature is similar to those observed after 3 h or 20.5 h at this temperature. Apparently, the cascade, involving two sequential cyclizations, is complete after ~2 h.

**Scheme 5:**
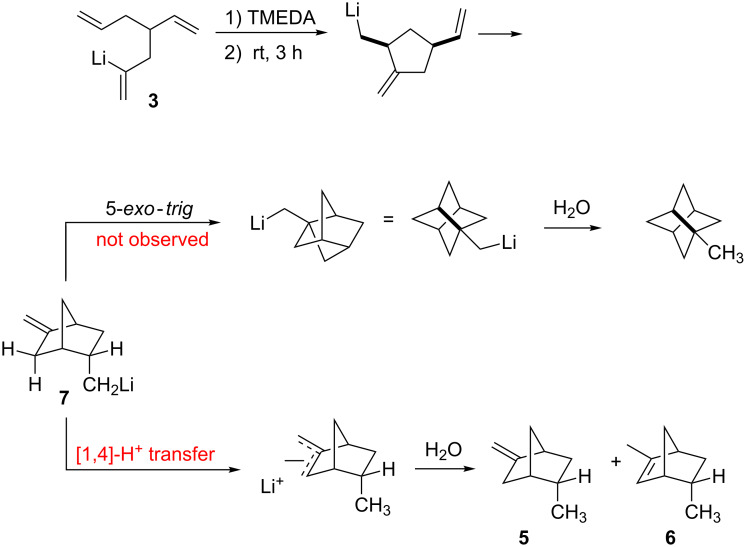
Proton transfer that foils final cyclization.

It would seem that a final cyclization to give 1-methylstellane is foiled by a formal [1,4]-proton transfer as depicted in Scheme 5. Cyclization of **3** quickly generates the monocyclic product and a second cyclization gives the *endo*-5-methyl-2-methylene organolithium **7** in nearly 90% yield. However, a proton transfer to give the more stable allylic anion apparently foils the final ring closure. Quenching of the reaction mixture then affords **5** and **6** in an approximate ratio of 2:1. In this connection, it should be noted that a 2-methylene-substituted bicyclo[2.2.1]heptane, such as product **5**, is known to be more stable than the isomeric norbornene, such as **6** [21]. Clearly, the energy required for the final 5-*exo*-*trig* cyclization to give the stellane framework is far greater than that required for the formal [1,4]-proton transfer that terminates the cascade.

**Scheme 6:**
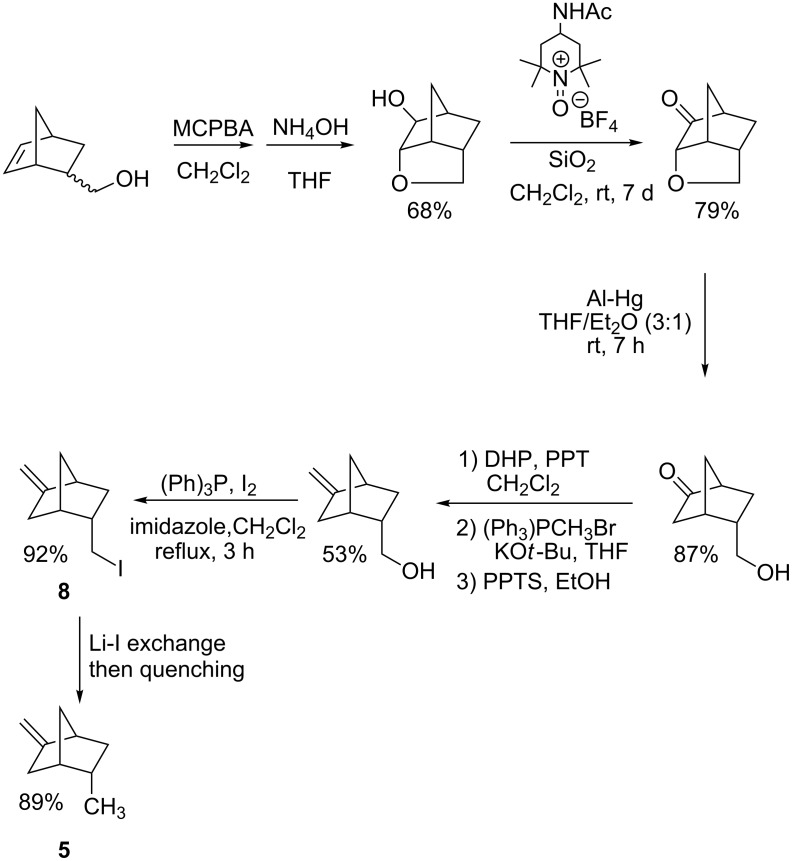
Preparation of iodide **7** and an authentic sample of **5**.

In an effort to further elucidate the nature of the proton transfer that terminates the cascade, *endo*-5-iodomethyl-2-methylenebicyclo[2.2.1]heptane (**8**) was prepared as illustrated in Scheme 6 (for details, see Supporting Information File 1). Iodide **8** was converted to the corresponding alkyllithium by low temperature lithium–iodine exchange in *n*-C_5_H_12_/Et_2_O (9:1 v/v) following our general protocol [22]. The exchange reaction is quite efficient as evidenced by the fact that quenching of a reaction mixture with MeOH affords an authentic sample of **5** in 89% isolated yield.

**Scheme 7:**
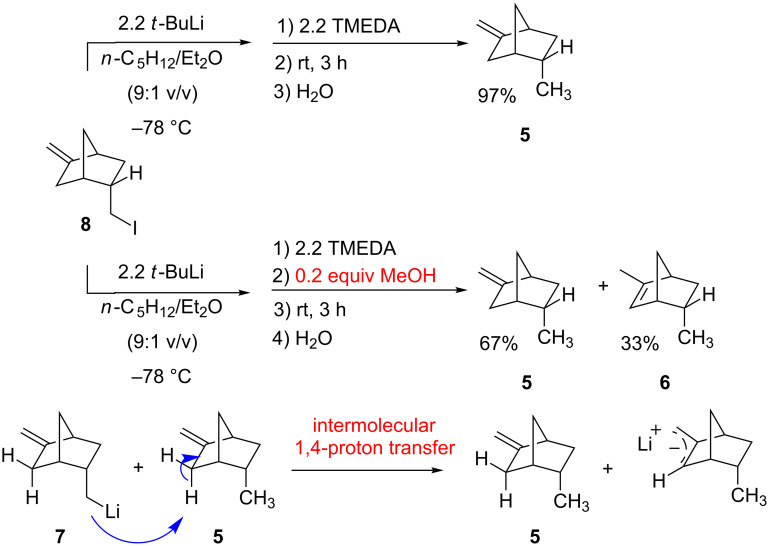
Evidence for the intermolecular nature of the formal [1,4]-proton transfer.

A series of experiments, involving warming solutions of alkyllithium **7** in scrupulously dry and oxygen-free pentane/Et_2_O containing 2.2 molar equiv of TMEDA for 3 h at room temperature, gave no evidence of the expected [1,4]-proton transfer, nor was there any evidence of 1-methylstellane: as illustrated at the top of Scheme 7, the exclusive product from such reactions, following quenching with water, was **5** isolated in 97% yield; there was no trace of **6** in any of the samples (for details, see Supporting Information File 1, p. S15). The failure to observe any rearranged product when alkyllithium **7** was warmed at room temperature was cause for initial concern since the result seemed to indicate that the proton transfer depicted in Scheme 5 is not a viable process. However, upon further consideration, it became apparent that the absence of **6** as a product from these reactions was an indication that the proton transfer is not an intramolecular process.

The intermolecular nature of the proton transfer is strongly supported by the following observation (Scheme 7): proton transfer is only observed when a small amount of **5** is present in the reaction mixture. Thus, the addition of a small quantity (0.2 molar equiv) of oxygen-free MeOH at −78 °C to a solution of bicyclic alkyllithium **7** served to generate a correspondingly small quantity of alkene **5**. As depicted in Scheme 7, when such a reaction mixture is allowed to stand at room temperature for 3 h in the presence of TMEDA, both **5** and **6**, in a ratio of 2:1, were produced after quenching with water. It would seem, as illustrated in Scheme 7, that the proton transfer is an *intermolecular* process catalyzed by a small quantity of *endo*-5-methyl-2-methylenebicyclo[2.2.1]heptane (**5**) present in reaction mixtures as a consequence of inadvertent quenching of **7** by solvent or adventitious acid during prolonged reaction times at room temperature.

## Conclusion

In retrospect, the failure to access the highly strained 1-methylstellane framework from an acyclic tri-olefinic vinyllithium (**3**) by sequential 5-*exo-trig* cyclizations is perhaps not surprising. The results of these studies do, however, serve to define a limit to the strain that may be accommodated by intramolecular carbolithiation. Moreover, it is significant that the penultimate olefinic alkyllithium **7** generated in the cascade appears to be resistant to rearrangement by proton transfer in the absence of a catalytic quantity of the hydrocarbon formed upon quenching of **7**. In short, 5-*exo-trig* carbolithiations are robust processes that are much more energetically favorable than are potential intramolecular proton transfers that would compromise such chemistry.

## Supporting Information

File 1 Experimental details and procedures for the preparation of all previously unreported compounds.
