# Supplementary material for: Intramolecular carbolithiation cascades as a route to a highly strained carbocyclic framework: competition between 5-exo-trig ring closure and proton transfer
Source: Beilstein J Org Chem. 2013 Mar 14;9:537–43. doi: 10.3762/bjoc.9.59 (PMC3628773; doi:10.3762/bjoc.9.59)
Supplement: File 1 — Experimental details and procedures for the preparation of all previously unreported compounds. [file Beilstein_J_Org_Chem-09-537-s001.pdf]

Supporting Information

for

Intramolecular carbolithiation cascades as a route to a highly strained carbocyclic framework: competition between 5-*exo-trig* ring closure and proton transfer

William F. Bailey*[1] and Justin D. Fair[2]

Address: [1] Department of Chemistry, University of Connecticut, Storrs, Connecticut 06269-3060, USA and [2] Department of Chemistry, Indiana University of Pennsylvania, Indiana, PA 15705, USA

Email: William F. Bailey - William.Bailey@uconn.edu

* Corresponding author

Experimental details and procedures for the preparation of all previously unreported compounds.

General Procedures. Spectroscopic and chromatographic procedures, methods used for the purification of reagents and solvents, as well as precautions regarding the manipulation of organolithiums have been previously described [1]. Organolithiums were filtered through a plug of dry glass wool under an atmosphere of argon before being titrated immediately before use with a standard solution of 2-butanol in xylenes using 1,10-phenanthroline in benzene as an indicator following the method described by Watson and Eastham [2]. All reactions involving alkyllithiums were performed in flame-dried glassware using standard syringe/cannula techniques under an atmosphere of dry, oxygen-free argon [3]. Dry, unsaturate-free *n*-pentane was obtained by repeated washings of commercial *n*-pentane with concentrated sulfuric acid until the acid layer remained clear, followed by washing with water and saturated aqueous sodium bicarbonate, drying over $MgSO_4$, and distillation of the purified pentane under nitrogen from a dark blue-purple solution of sodium / benzophenone/tetraglyme. Anhydrous diethyl ether (J. T. Baker) and tetrahydrofuran (J. T. Baker) were distilled from dark blue-purple solutions of sodium and benzophenone. TMEDA was distilled under nitrogen from calcium hydride immediately before use. Anhydrous MeOH and MeOD were stored over 4 Å molecular sieves and were rendered essentially oxygen free before use by bubbling dry, deoxygenated argon gas through the neat liquid for at least 5 min before use: failure to follow this rather stringent protocol was found to result in consumption of organolithiums through rapid reaction with adventitious oxygen.

Low-temperature reactions requiring either precise temperature control or long reaction times were performed utilizing a Neslab Cryotrol temperature control cooling bath. Gas–liquid chromatography (GC) was performed on a Hewlett-Packard 5890 gas chromatograph equipped with a flame-ionization detector and fitted with a 25 m x 0.20 mm, 0.33 μm DB–5MS (5% diphenyl/95% dimethylpolysiloxane) fused silica glass capillary column (J & W Scientific). The nitrogen carrier gas was maintained at an average 16.0 cm/s linear velocity corresponding to an outlet flow of 0.3 mL/min, a 10.0 psi inlet pressure, and a holdup time of 2.60 min. The split ratio was set at 60:1, corresponding to a 19.8 mL/min split vent flow. Gas–liquid chromatography–mass spectrometry (GC–MS) was performed on a Hewlett-Packard 5890 gas chromatograph fitted with a 25 m x 0.20 mm, 0.33 μm DB–5MS (5% diphenyl/95% dimethylpolysiloxane) fused silica glass capillary column (J & W Scientific) and interfaced with a Hewlett-Packard 5971 mass selective detector (electron impact, EI). The helium carrier gas was maintained at an average 30.0 cm/s linear velocity corresponding to a vacuum outlet flow of 0.5 mL/min, a 11.9 psi inlet pressure, and a holdup time of 1.39 min. The split ratio was set at 60:1, corresponding to a 33.0 mL/min split vent flow. Preparative gas chromatography was performed on a Varian Aerograph A-90P equipped with a thermal conductivity detector and fitted with one of the following packed columns: 10 ft, 10 % SE-30 on Anakrom A (60/80 mesh), 10 ft, 5% QF1 on Chromosorb A (60/80 mesh), or a 10 ft silver nitrate on silica (230–325 mesh, 44–63 mm). The average linear flow for the 4 mm i.d.,

0.25 in o.d. packed columns was measured to be 510 cm/s (flow rate = 43 mL/min).

^{1}H and ^{13}C NMR spectra were acquired on a Bruker DRX-300 or a Bruker DRX-400 spectrometer using $CDCl_3$ as solvent unless otherwise specified. Proton and carbon spectra were referenced at δ = 7.26 and 77.23, respectively, for the residual ^{1}H resonance of the solvent and the center line of the ^{13}C absorption of $CDCl_3$ and are reported relative to Me_4Si at δ = 0.00.

tert-Butyl 2-allyl-4-bromo-4-pentenoate

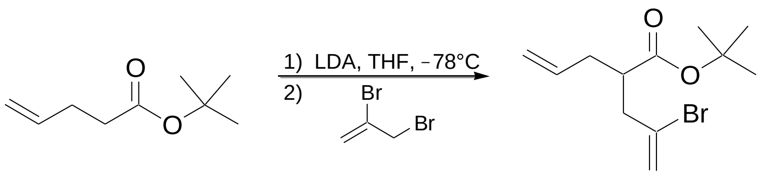

A solution of 13.29 g (131.4 mmol) of diisopropylamine in 250 mL of dry tetrahydrofuran was cooled to –78 °C, and 71.8 mL of a 1.69 M solution of *n*-butyllithium in hexane (121 mmol) was added dropwise. The reaction was stirred for 20 min followed by dropwise addition of a solution of 15.78 g (101.1 mmol) of *tert*-butyl 4-pentenoate [4] in 25 mL of dry tetrahydrofuran. The resulting solution was stirred at –78 °C for an additional 20 min before addition of 22.0 g (111 mmol) of 2,3-dibromopropene [5] and 18.2 g (101 mmol) of hexamethylphosphoramide. The reaction mixture was stirred at –78 °C for 1 h and then allowed to warm and stand at room temperature for 3.5 h before the addition of 275 mL of saturated, aqueous ammonium chloride. The reaction

mixture was transferred to a separatory funnel, the organic layer was separated and concentrated, and the aqueous layer was extracted with 100 mL ether. The combined organic layers were washed sequentially with water, 10% aqueous hydrochloric acid, saturated, aqueous sodium bicarbonate, and brine. The ethereal layer was dried ($MgSO_4$) and concentrated by rotary evaporation, and the residue was distilled (Kugelrohr) to afford 23.1 g (83%) of the ester; bp 180 °C (5 mm); ^{1}H NMR (400 MHz, $CDCl_3$) δ 1.44 (s, 9 H), 2.40 – 2.21 (m, 2H), 2.53 – 2.46 (m, 1 H), 2.78 – 2.70 (m, 2 H), 5.11 – 5.04 (m, 2 H), 5.45 (d, 1 H, *J* = 1.6 Hz), 5.62 (m, 1 H), 5.78 – 5.68 (m, 1 H); ^{13}C NMR (100 MHz, $CDCl_3$) δ 28.3, 35.9, 43.2, 44.3, 81.0, 117.5, 118.8, 131.7, 134.9, 173.5; anal. calcd for $C_{12}H_{19}BrO_2$: C. 52.38; H, 6.96; found: C, 51.99; H, 6.58.

2-Allyl-4-bromo-4-penten-1-ol

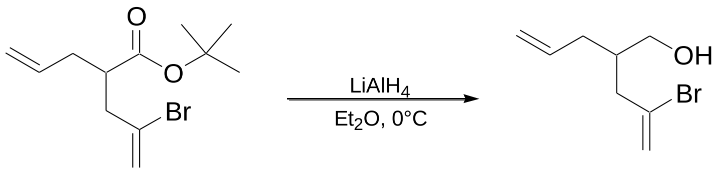

A slurry of 12.41 g (327.1 mmol) of lithium aluminium hydride in 500 mL of anhydrous diethyl ether was stirred at 0 °C, and a solution of 30.00 g (121 mmol) of *tert*-butyl 2-allyl-4-bromo-4-pentenoate in 20 mL diethyl ether was added dropwise. The reaction mixture was stirred for 2 h at room temperature and then hydrolyzed by sequential, dropwise addition of 24.8 mL of water, 24.8 mL of 15% aqueous sodium hydroxide, and 74.4 mL of water. The resulting mixture was filtered and the solid

was extracted overnight in a Soxhlet apparatus. The combined extract and organic layer were combined and concentrated by rotary evaporation, and the residue was taken up in 100 mL of diethyl ether and washed with four 50 mL portions of water. The ethereal layer was dried ($MgSO_4$), concentrated by rotary evaporation, and the residue was distilled (Kugelrohr) to afford 15.6 g (70%) of the alcohol; bp 130 °C (5 mm): ^{1}H NMR (400 MHz, $CDCl_3$) δ 1.37 (br s, 1 H), 2.08 – 1.99 (m, 1 H), 2.18 – 2.14 (m, 2 H), 2.41 (ddd, 1 H, *J* = 1.0 Hz, *J* = 7.1 Hz, *J* = 14.5 Hz), 2.53 (ddd, 1 H, *J* = 1.0 Hz, *J* = 7.1 Hz, *J* = 14.5 Hz), 3.61 (apparent d, 2 H, *J* = 4.9 Hz), 5.12 – 5.05 (m, 2 H), 5.47 (d, 1 H, *J* = 1.5 Hz), 5.62 (dd, 1 H, *J* = 1.1 Hz, *J* = 2.6 Hz), 5.87 – 5.76 (m, 1 H); ^{13}C NMR (100 MHz, $CDCl_3$) δ 35.0, 38.7, 43.0, 64.3, 117.2, 118.6, 133.2, 136.4; anal. calcd for $C_8H_{13}BrO$: C. 46.85; H, 6.39; found: C, 46.54; H, 6.06.

2-Bromo-4-vinyl-1,6-heptadiene (2)

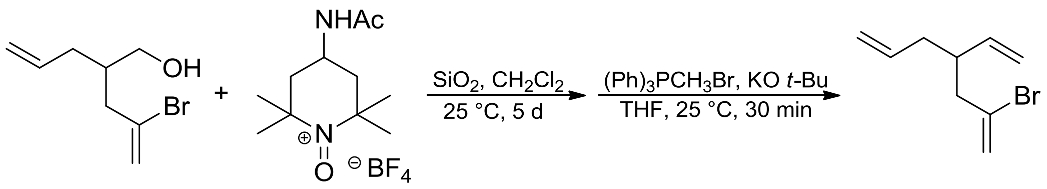

A mixture of 15.50 g (75.98 mmol) of 2-allyl-4-bromo-4-penten-1-ol, 25.13 g (83.50 mmol) of 4-acetamido2,2,6,6-tetramethylpiperidine-1-oxoammonium tetrafluoroborate, and 25 g of silica gel in 125 mL of methylene chloride was stirred at room temperature for 5 d until a potassium iodide test proved negative for the oxidant. The reaction mixture was flushed through a 100 g pad of silica gel with four

column volumes of methylene chloride. The filtrate was dried (Na_2SO_4) and concentrated, and the residue was used without further purification.

A 500 mL, flame-dried, round-bottomed flask, was charged with 325 mL of dry tetrahydrofuran, 37.22 g (104.2 mmol) of methyltriphenylphosphonium bromide, and 11.70 g (104.2 mmol) of potassium *tert*-butoxide. The resulting bright yellow suspension was stirred for 30 min before being added dropwise via cannula to a solution of the crude aldehyde in 75 mL of dry tetrahydrofuran. The reaction mixture was stirred for 2 h before the addition of 400 mL of saturated, aqueous ammonium chloride. The organic layer was removed and concentrated, the aqueous layer was extracted with three 20 mL portions of pentane, and the combined extracts were diluted with 50 mL of pentane, and washed sequentially with two 20 mL portions each of water and brine. The organic layer was passed through a 100 g pad of silica gel with four column volumes of pentane, dried ($MgSO_4$), and concentrated to afford 12.40 g (83 %) of 2-bromo-4-vinyl-1,6-heptadiene: ^{1}H NMR ($CDCl_3$) δ 2.23 – 2.05 (m, 2 H), 2.40 – 2.33 (m, 1 H), 2.58 – 2.46 (m, 2 H), 5.08 – 5.01 (m, 4H), 5.43 (d, 1 H, J = 1.5 Hz), 5.66 – 5.54 (m, 2 H), 5.82 – 5.71 (m, 1 H); ^{13}C NMR (100 MHz, $CDCl_3$) δ 38.4, 41.4, 46.2, 115.6, 116.8, 118.3, 133.0, 136.3, 140.5; HRMS-CI (*m*/*z*): $[M + H]^+$ calcd for $C_9H_{14}{}^{81}Br$: 203.02584; found, 203.02603.

4-Vinyl-1,6-heptadiene (4)

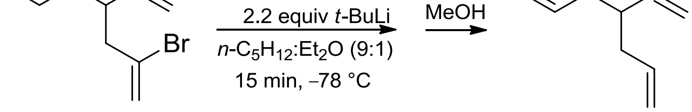

A stirred solution of 0.20 g (1.00 mmol) of 2-bromo-4-vinyl-1,6-heptadiene in 9 mL of dry *n*-pentane and 1 mL of dry diethyl ether was cooled to –78 °C under an atmosphere of argon, and 1.31 mL (2.20 mmol) of a 1.68 M solution of *tert*-butyllithium in pentane was added dropwise. The solution was stirred at –78 °C for 15 min before the dropwise addition of 1 mL of deoxygenated methanol. The organic layer was separated and washed with water and brine, and then dried ($MgSO_4$) and concentrated to afford 120 mg (98%) of the title compound: ^{1}H NMR (300 MHz, $CDCl_3$) δ 2.12 – 2.02 (m, 2 H), 2.22 – 2.12 (m, 3 H), 5.04 – 4.95 (m, 6 H), 5.81 – 5.60 (m, 3 H); ^{13}C NMR (100 MHz, $CDCl_3$) δ 38.8, 43.4, 114.6, 116.2, 137.0, 142.1; HRMS–FAB (*m*/*z*): [M + H] calcd for C_9H_{14}, 123.1176; found, 123.1184.

4-Oxatricyclo[4.2.1.0^{3,7}]nonan-2-ol

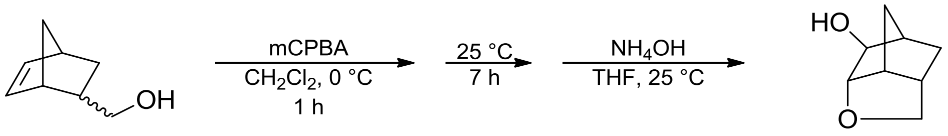

The title compound was prepared by modification of a standard procedure [6]. Thus, a solution of 11.35 g (82.17 mmol) of *m*-chloroperoxybenzoic acid in 80 mL of methylene chloride was cooled to 0 °C and a solution of 6.00 g (48.35 mmol) of 5-norbornene-2-methanol (Aldrich, 77% *endo*-isomer) in 20 mL of methylene chloride was added dropwise. The reaction mixture was stirred for 1 h at 0 °C and then at 25 °C for 7 h before addition of 20 mL of saturated, aqueous sodium sulfite. The resulting mixture was passed through a bed of Celite and the filtrate was washed with saturated, aqueous sodium bicarbonate and brine, and then concentrated. The

thick, opaque oil was taken up in 200 mL of tetrahydrofuran and 15 mL of concentrated, aqueous sodium hydroxide. The resulting solution was stirred for 8 h at room temperature before the addition of 200 mL of saturated, aqueous ammonium chloride. The resulting solution was acidified using 10% aqueous, hydrochloric acid to a pH ~8, the layers were separated, and the aqueous layer was extracted with three 100 mL portions of methylene chloride. The organic layers were combined, concentrated, taken up in 150 mL of diethyl ether, washed with brine, dried ($MgSO_4$), and concentrated to give 4.63 g (68%) of the title compound [7]: ^1H NMR (300 MHz, $CDCl_3$) δ 0.96 (dt, 1 H, *J* = 2.1 Hz, *J* = 12.9 Hz), 1.48 (dd, 1 H, *J* = 1.2 Hz, *J* = 10.6 Hz), 1.84 (m, 1 H), 1.95 (d, 1 H, *J* = 10. 7 Hz), 2.07 (m, 1 H), 2.28 (m, 1 H), 2.58 (m, 1 H), 2.85 (bs, 1 H), 3.44 (s, 1 H), 3.62 (d, 1 H, *J* = 8.0 Hz), 3.77 (dd, 1 H, *J* = 4.0 Hz, *J* = 7.9 Hz), 3.93 (d, 1 H, *J* = 4.4 Hz); ^{13}C NMR (100 MHz, $CDCl_3$) δ 33.7, 34.0, 37.5, 41.4, 44.8, 75.4, 81.4, 87.9.

4-Oxatricyclo[4.2.1.0^{3,7}]nonan-2-one

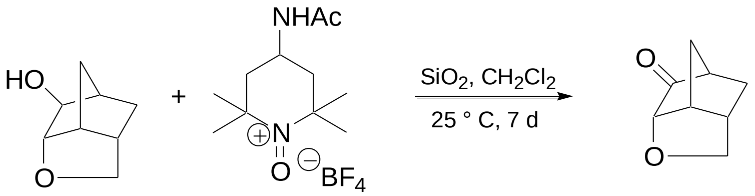

A slurry of 6.84 g (48.8 mmol) of 4-oxatricyclo[4.2.1.0^{3,7}]nonan-2-ol, 20.51 g (68.33 mmol) of 4-acetamido2,2,6,6-tetramethylpiperidine-1-oxoammonium tetrafluoroborate, and 68 g of silica gel in 500 mL of methylene chloride was stirred at 25 °C for 7 d until a potassium iodide test proved negative for the oxidant. The reaction mixture was flushed through a short pad of silica gel with four column

volumes of methylene chloride. The filtrate was concentrated to afford 5.26 g (79.1%) of the known ketone as a gelatinous, opaque solid [7]: ^{1}H NMR (300 MHz, $CDCl_3$) δ 1.33 (d, 1 H, *J* = 13.1 Hz), 1.76 (s, 2 H), 2.03 (m, 1 H), 2.45 (m, 1 H), 2.66 (m, 1 H), 2.99 (m, 1 H), 3.74 (d, 1 H, *J* = 8.4 Hz), 3.82 (d, 1H, *J* = 5.3 Hz), 3.99 (dd, 1 H, *J* = 4.5 Hz, *J* = 8.4 Hz); ^{13}C NMR (300 MHz, $CDCl_3$) δ 30.4, 33.1, 37.4, 44.5, 45.7, 76.2, 81.0, 214.1.

***endo*-5-Hydroxymethylbicyclo[2.2.1]heptan-2-one**

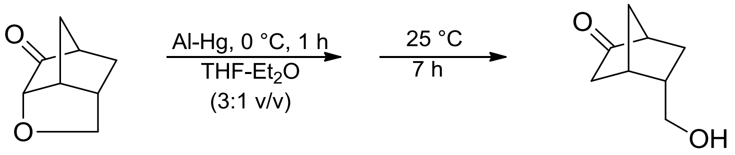

The title compound was prepared using the procedure of Arai and co-workers [8]. Thus, a solution of 2.67 g (19.3 mmol) of 4-oxatricyclo[4.2.1.0^{3,7}]nonan-2-one in 100 mL of a tetrahydrofuran – diethyl ether (3:1 by vol) was cooled to 0 °C before the portion-wise addition of 8.0 g of freshly prepared Al–Hg amalgam [9]. The reaction mixture was stirred for 1 h at 0 °C and then warmed to room temperature and stirred at 25 °C for an additional 7 h. The suspension was filtered through a bed of Celite using six column volumes of ethyl acetate, and the filtrate was dried ($MgSO_4$) and concentrated to afford 2.37 g (87 %) of the title compound as a clear oil: ^{1}H NMR (300 MHz, $CDCl_3$) δ 1.03 – 0.98 (m, 1 H), 1.41 – 1.39 (m, 1 H), 1.79 – 1.65, (m, 2 H), 2.06 – 1.91 (m, 2 H), 2.18 – 2.11 (m, 1 H), 2.39 – 2.25 (m, 1 H), 2.55 (d, 1 H, *J* = 4.8 Hz), 2.70 (bs, 1 H), 3.49 – 3.42 (m, 1 H), 3.64 – 3.58 (m, 1 H); ^{13}C NMR (100 MHz, $CDCl_3$) δ 28.4, 37.1, 38.8, 39.5, 40.7, 50.4, 63.0, 218.3.

(*endo*-5-Methylenebicyclo[2.2.1]-2-heptyl)methanol

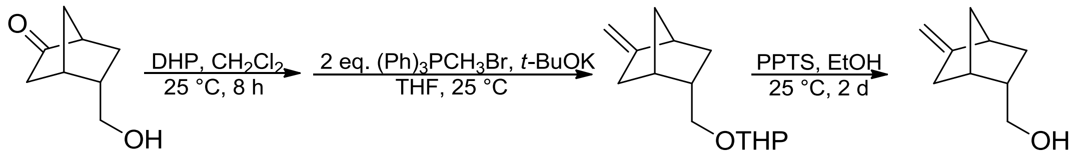

A solution of 0.50 g (3.57 mmol) of *endo*-5-hydroxymethylbicyclo[2.2.1]heptan-2-one, 0.45 g (5.36 mmol) of dihydropyran, and 0.09 g (3.57 mmol) of pyridinium *p*-toluenesulfonate in 24 mL of dry methylene chloride was stirred for 4 h at room temperature. The solution was then diluted with 150 mL of diethyl ether and washed once with brine, dried (Na_2SO_4), and concentrated. The product was used without further purification.

A 100 mL, flame-dried, round-bottomed flask was charged with 50 mL of dry tetrahydrofuran, 1.40 g (3.93 mmol) of methyltriphenylphosphonium bromide, and 0.44 g (3.93 mmol) of potassium *tert*-butoxide. The resulting bright yellow solution was stirred for 30 min before being added dropwise via cannula to a solution of the crude ketone in 50 mL of dry tetrahydrofuran. The reaction mixture was stirred for 30 min before the addition of 35 mL of saturated, aqueous ammonium chloride. The organic layer was removed, the aqueous layer was extracted with three 20 mL portions of pentane, and the combined extracts were diluted with 50 mL of pentane and washed sequentially with two 20 mL portions of water and brine. Column chromatography on silica gel using diethyl ether/pentane (10:90 v/v) gave 480 mg (60%) of the tetrahydropyranyl-protected alkenol.

A solution of 200 mg (0.90 mmol) of the tetrahydropyranyl-protected alkenol in 10 mL of ethanol containing a catalytic quantity of pyridinium *p*-toluenesulfonate was

stirred overnight at 55 °C. Solvent was removed and the residue was purified by column chromatography on silica gel using diethyl ether – hexane (30/70 v/v; R_f = 0.26) to afford 110 mg (88%) of the title compound as a clear oil (an overall yield of 53% from *endo*-5-hydroxymethylbicyclo[2.2.1]heptan-2-one): ^{1}H NMR (300 MHz, $CDCl_3$) δ 0.76 (dq, 1 H, *J* = 2.0 Hz, *J* = 12.0 Hz), 1.46 – 1.36 (m, 2 H), 1.80 (dt, 1 H, *J* = 4.4 Hz, *J* = 11.8 Hz), 2.19 – 1.95 (m, 3 H), 2.36 (s, 1 H), 2.60 (d, 1 H, *J* = 3.9 Hz), 2.75 (bs, 1 H), 3.46 – 3.40 (m, 1 H), 3.56 – 3.50 (m, 1 H), 4.52 (s, 1 H), 4.75 (s, 1 H); ^{13}C NMR (100 MHz, $CDCl_3$) δ 31.9, 34.1, 38.5, 40.5, 41.6, 45.9, 64.6, 101.8, 155.2; ^{13}C DEPT NMR (100 MHz, $CDCl_3$) δ 31.9 (CH_2), 34.1 (CH_2), 38.5 (CH), 40.5 (CH_2), 41.6 (CH), 45.9 (CH), 64.6 (CH_2), 101.8 (CH_2); HRMS–ES (*m*/*z*): [M – H] calcd for $C_9H_{13}O$, 137.097; found, 137.098.

endo-5-Iodomethyl-2-methylenebicyclo[2.2.1]heptane (8)

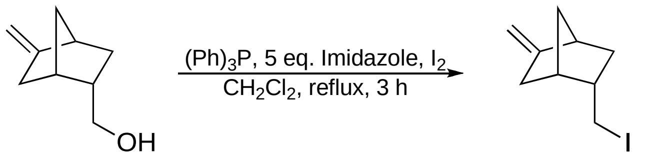

A mixture of 390 mg (5.58 mmol) of imidazole and 570 g (2.17 mmol) of triphenylphosphine in 30 mL dry methylene chloride was stirred at room temperature for 30 min until dissolution was complete, and 550 mg (2.17 mmol) of iodine was then added. The resulting solution was stirred for 10 min at room temperature before the addition of 200 mg (1.45 mmol) of (*endo*-5-methylenebicyclo[2.2.1]-2-heptyl)methanol in 5 mL methylene chloride. The reaction mixture was heated under reflux for 3 h under an atmosphere of argon, cooled to room temperature, and

1 g of silica gel was added before concentration of the reaction mixture. The silica gel was flushed with 20 mL of pentane, and the eluant was concentrated and dried (Na_2SO_4) to afford 330 mg (92%) of the iodide as a clear oil: ^{1}H NMR (300 MHz, $CDCl_3$) δ 0.87 (dq, 1 H, *J* = 2.4 Hz, *J* = 12.4 Hz), 1.58 – 1.47 (m, 2 H), 2.07 – 1.93 (m, 2 H), 2.17 (s, 2 H), 2.45 – 2.42 (m, 1 H), 2.82 – 2.81 (m, 1 H), 3.10 – 3.03 (m, 1 H), 3.29 – 3.23 (m, 1 H), 4.60 (s, 1 H), 4.82 (s, 1 H); ^{13}C NMR (100 MHz, $CDCl_3$) δ 10.9, 31.2, 38.6, 40.9, 42.0, 42.8, 47.5, 102.6, 154.6; HRMS–EI (70 eV) *m/z*: M^+ calcd for $C_9H_{13}I$, 248.0062; found, 248.0059.

endo-5-Methyl-2-methylenebicyclo[2.2.1]heptane (5)

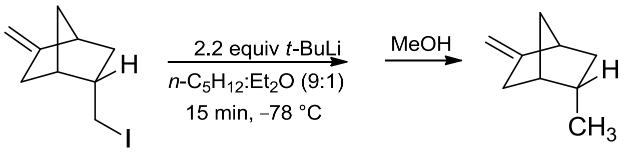

A stirred solution of 230 mg (0.93 mmol) of *endo*-5-iodomethyl-2-methylenebicyclo[2.2.1]heptane (**8**) in 9 mL of dry *n*-pentane and 1 mL of dry diethyl ether was the cooled to –78 °C under an atmosphere of argon and 1.98 mL (2.04 mmol) of a 1.03 M solution of *tert*-butyllithium in pentane was added dropwise. The solution was stirred at –78 °C for 15 min before the dropwise addition of 1 mL of deoxygenated methanol. The organic layer was washed with water and brine, and concentrated and dried ($MgSO_4$) to afford 110 mg (89%) of **5** as a clear oil: ^{1}H NMR (400 MHz, $CDCl_3$) δ 0.73 – 0.77 (m, 1 H), 0.98 (d, 3 H, *J* = 6.8 Hz), 1.42 – 1.46 (m, 2 H), 1.92 – 2.02 (m, 3 H), 2.14 (s, 1 H), 2.24 – 2.34 (m, 1 H), 2.60 – 2.61 (m, 1 H), 4.55 (s, 1 H), 4.78 (s, 1 H); ^{13}C NMR (100 MHz, $CDCl_3$) δ 17.7, 31.6, 33.5, 38.9,

41.2, 42.3, 47.2, 101.1, 156.5; HRMS–EI (70 eV) *m/z*: M^+ calcd for C_9H_{14}, 122.1096; found, 122.1097.

General procedure (Table 1 and Scheme 4) for cyclization of the vinyllithium (3) derived from 2-bromo-4-vinyl-1,6-heptadiene (2): Preparation of *endo*-5-methyl-2-methylenebicyclo[2.2.1]heptane (5) and *endo*-2,5-dimethyl-bicyclo[2.2.1]hept-2-ene (6)

A stirred solution of 200 mg (1.00 mmol) of 2-bromo-4-vinyl-1,6-heptadiene (**2**) in 9 mL of dry *n*-pentane and 1 mL of dry diethyl ether was cooled to –78 °C under an atmosphere of argon, and 1.31 mL (2.20 mmol) of a 1.68 M solution of *tert*-butyllithium in pentane was added dropwise. The solution was stirred for 15 min at –78 °C before the dropwise addition of 0.26 g (2.20 mmol) of dry, oxygen-free TMEDA. After 15 min the dry ice–acetone cooling bath was removed, and stirring was continued for 3 h as the reaction mixture warmed and was allowed to stand at room temperature before the addition of 1 mL of deoxygenated water. The reaction mixture was washed with water and brine, and dried (Na_2SO_4) and concentrated to give a clear oil. The residual oil was flushed through a short pad of silica gel with pentanes to afford 100 mg (82%) of a mixture of two bicyclic alkenes. Analysis by GC/FID [5% diphenyl / 95% dimethylpolysiloxane, 25 m x 0.20 mm, 0.33 μm df; initial temperature of 45 °C for 10 min, 2 °C/min temperature ramp to 56 °C, 15 °C/min temperature ramp to 240 °C, final time of 5 min] revealed that the mixture was composed of 4.8% 4-vinyl-1,6-heptadiene (**4**), 31.8% of *endo*-2,5-dimethylbicyclo[2.2.1]hept-2-ene (**6**) and 63.4% of *endo*-5-methyl-2-

methylenebicyclo[2.2.1]heptane (**5**). Preparative GC (QF-1, 60 °C, isothermal conditions) allowed isolation of the known **6**: ^{1}H NMR (400 MHz, $CDCl_3$) δ 0.37 – 0.42 (m, 1 H), 0.77 (d, 3 H, *J* = 6.9 Hz), 0.88 (t, 1 H, *J* = 7.1 Hz), 1.26 – 1.32 (m, 1 H), 1.39 – 1.42 (m, 1 H), 1.75 (d, 3 H, *J* = 1.54 Hz), 1.84 (m, 1 H), 2.50 (m, 2 H), 5.49 (s, 1 H); ^{13}C NMR (100 MHz, $CDCl_3$) δ 15.2, 19.7, 33.7, 35.0, 48.0, 48.1, 49.9, 125.3, 147.2. The remaining two components (**4** and **5**) were identical in all respects to authentic samples as described above.

Attempted cyclization of the organolithium derived from *endo*-5-iodomethyl-2-methylenebicyclo[2.2.1]heptane (8).

A stirred solution of 250 mg (1.00 mmol) of *endo*-5-iodomethyl-2-methylenebicyclo[2.2.1]heptane (**8**) in 9 mL of dry *n*-pentane and 1 mL of dry diethyl ether was cooled to –78 °C under an atmosphere of argon, and 0.76 mL (2.20 mmol) of a 2.88 M solution of *tert*-butyllithium in pentane was added dropwise. The solution was stirred for 15 min at –78 °C before the dropwise addition of 0.26 g (2.20 mmol) of dry, oxygen-free TMEDA. After 15 min, the dry ice–acetone cooling bath was removed, and stirring was continued for 3 h as the reaction mixture warmed and was then allowed to stand at room temperature before the addition of 1 mL of deoxygenated water. The reaction mixture was washed with water and brine, and dried (Na_2SO_4) and concentrated to give 120 mg (97%) of *endo*-5-methyl-2-methylenebicyclo[2.2.1]heptane (**5**) as a clear oil. Analysis by GC/FID [5% diphenyl / 95% dimethylpolysiloxane, 25 m x 0.20 mm, 0.33 μm df; initial temperature of 45 °C

for 10 min, 2 °C/min temperature ramp to 56 °C, 15 °C/min temperature ramp to 240 °C, final time of 5 min] revealed that the oil was pure **5**.

Rearrangement (Scheme 7) of the organolithium (7) derived from *endo*-5-iodomethyl-2-methylenebicyclo[2.2.1]heptane (8).

A stirred solution of 250 mg (1.00 mmol) of *endo*-5-iodomethyl-2-methylenebicyclo[2.2.1]heptane (**8**) in 9 mL of dry *n*-pentane and 1 mL of dry diethyl ether was cooled to –78 °C under an atmosphere of argon and 0.76 mL (2.20 mmol) of a 2.88 M solution of *tert*-butyllithium in pentane was added dropwise. The solution was stirred for 15 min at –78 °C before the dropwise addition of 0.26 g (2.20 mmol) of dry TMEDA. After 15 min, 8.0 μL (0.20 mmol) of deoxygenated MeOH was added using a 10 μL syringe, the dry ice-acetone cooling bath was removed, and the reaction mixture was allowed to stand at room temperature for 3 h before addition of 1.0 mL of oxygen-free water. The organic layer was washed with water and brine, and dried (Na_2SO_4) and concentrated to give 110 mg (89%) of a clear oil. Analysis by GC/FID [5% diphenyl / 95 %dimethylpolysiloxane, 25 m x 0.20 mm, 0.33 μm df; initial temperature of 45 °C for 10 min, 2 °C/min temperature ramp to 56 °C, 15 °C/min temperature ramp to 240 °C, final time 5 min] revealed that the mixture was composed of 33% of *endo*-2,5-dimethylbicyclo[2.2.1]hept-2-ene (**6**) and 67 % of *endo*-5-methyl-2-methylenebicyclo[2.2.1]heptane (**5**).

References

1. Bailey, W. F.; Khanolkar, A. D.; Gavaskar, K.; Ovaska, T. V.; Rossi, K.; Thiel, Y.; Wiberg, K. B. *J. Am. Chem. Soc*. **1991**, *113*, 5720.
2. Watson, S. C.; Eastham, J. F. *J. Organomet. Chem.* **1967**, *9*, 165.
3. Shriver, D. F.; Drezdon, M. A. *The Manipulation of Air-Sensitive Compounds;* 2nd ed.; John Wiley & Sons: New York, 1986.
4. Harris, D, H; Herr, R. J.; Weinreb, S. M, *J. Org. Chem.* **1993**, 58, 5452.
5. Lespieau, R.; Bourguel, M. *Organic Syntheses. Coll. Vol. 1*, Wiley and Sons: New York, 1947, 209.
6. Furniss, B. S.; Hannaford, A. J.; Smith, P.G.; Tatchell, A.R. *Vogel's Textbook of Practical Organic Chemistry*, 5th Ed.; Prentice Hall: New York, 1989; p 1134.
7. Yamamoto, K.; Momose, S., Funanashi, M.; Ebata, S.; Ohmura, H.; Komatsu, H.; Miyazawa, M. *Chem. Lett.* **1994**, *2*, 189.
8. Arai, Y.; Kawanami, S.; Koizumi, T. *J. Chem. Soc., Perkin Trans. I* **1991**, 2969.
9. Fieser, L. F.; Fieser, M. *Reagents for Organic Synthesis*, Wiley and Sons: New York, 1967, p. 20.
10. Cohen, C. A. Cyclopentane-1,3-dicarboylic Acids. D. E. Patent 1092909, December 30, 1957
